# P12 Partial implementation of procalcitonin measurement as an antibiotic stewardship in the ICU

**DOI:** 10.1093/jacamr/dlad066.016

**Published:** 2023-06-14

**Authors:** Simon Gill, Ryan Phillips, David Cain, Neil Powell

**Affiliations:** Critical Care, Royal Cornwall Hospital, Truro TR1 3LJ, UK; Critical Care, Royal Cornwall Hospital, Truro TR1 3LJ, UK; Critical Care, Royal Cornwall Hospital, Truro TR1 3LJ, UK; Pharmacy Department, Royal Cornwall Hospital, Truro TR1 3LJ, UK

## Abstract

**Background:**

Procalcitonin (PCT) was introduced to the Royal Cornwall Hospital ICU in April 2020. A local PCT testing and decision support algorithm was developed using published consensus guidelines. We report our adherence to this algorithm in the ICU.

**Methods:**

This retrospective observational study included all patients discharged from ICU from 1 February 2022 to 28 February 2022. Patients who received antibiotics during their admission were identified. Data on antibiotic indication, antibiotic course length in the ICU and pre-ICU and PCT testing were collected and compared with local PCT-based antibiotic prescribing guideline.

**Results:**

Of 50 patients discharged, 34 (68%) received antibiotics and 26 for acute infections and 8 for deep-seated infections or surgical prophylaxis. A baseline PCT was sent in 10 (38%) patients with acute infections, with 4 having repeat PCT testing. Of four patients with a low baseline PCT, three were repeated and remained low. Two of these patients had antibiotics stopped as per PCT algorithm. A further patient with a low PCT was not initiated on antibiotics. Six patients had a high baseline PCT (>0.5) and one had a repeat PCT which remained high. Antibiotics were continued in all 6 patients. 16 patients with acute infections did not have baseline PCT testing with one having a random PCT during the antibiotic course. Five patients had deep-seated infections, and therefore, PCT testing was not indicated. However, 2 had baseline and repeat PCTs, with repeat PCTs low (<0.5) and antibiotics continued. Median course lengths were 5 days in the ICU and 6 days when pre-ICU antibiotics were included.

**Conclusions:**

PCT testing in our ICU is low with only a third of eligible patients having a baseline PCT test with repeat PCT testing in accordance with local guidelines in the minority of these. There is some limited evidence that PCT testing is impacting antibiotic decision making in our ICU. We have identified that antibiotic course lengths in our ICU are relatively short. We have introduced PCT measurement as part of antimicrobial stewardship in our ICU. Fully embedding PCT testing, and PCT-guided antibiotic decision making, may further reduce antibiotic use. How to achieve this is challenging—there is a paucity of published PCT implementation studies.

